# Oral Supplementation with Non-Absorbable Antibiotics or Curcumin Attenuates Western Diet-Induced Atherosclerosis and Glucose Intolerance in LDLR−/− Mice – Role of Intestinal Permeability and Macrophage Activation

**DOI:** 10.1371/journal.pone.0108577

**Published:** 2014-09-24

**Authors:** Siddhartha S. Ghosh, Jinghua Bie, Jing Wang, Shobha Ghosh

**Affiliations:** Department of Internal Medicine, Virginia Commonwealth University Medical Center, Richmond, Virginia, United States of America; Charité, Campus Benjamin Franklin, Germany

## Abstract

Association between circulating lipopolysaccharide (LPS) and metabolic diseases (such as Type 2 Diabetes and atherosclerosis) has shifted the focus from Western diet-induced changes in gut microbiota *per se* to release of gut bacteria-derived products into circulation as the possible mechanism for the chronic inflammatory state underlying the development of these diseases. Under physiological conditions, an intact intestinal barrier prevents this release of LPS underscoring the importance of examining and modulating the direct effects of Western diet on intestinal barrier function. In the present study we evaluated two strategies, namely selective gut decontamination and supplementation with oral curcumin, to modulate Western-diet (WD) induced changes in intestinal barrier function and subsequent development of glucose intolerance and atherosclerosis. LDLR−/− mice were fed WD for 16 weeks and either received non-absorbable antibiotics (Neomycin and polymyxin) in drinking water for selective gut decontamination or gavaged daily with curcumin. WD significantly increased intestinal permeability as assessed by *in vivo* translocation of FITC-dextran and plasma LPS levels. Selective gut decontamination and supplementation with curcumin significantly attenuated the WD-induced increase in plasma LPS levels (3.32 vs 1.90 or 1.51 EU/ml, respectively) and improved intestinal barrier function at multiple levels (restoring intestinal alkaline phosphatase activity and expression of tight junction proteins, ZO-1 and Claudin-1). Consequently, both these interventions significantly reduced WD-induced glucose intolerance and atherosclerosis in LDLR−/− mice. Activation of macrophages by low levels of LPS (50 ng/ml) and its exacerbation by fatty acids is likely the mechanism by which release of trace amounts of LPS into circulation due to disruption of intestinal barrier function induces the development of these diseases. These studies not only establish the important role of intestinal barrier function, but also identify oral supplementation with curcumin as a potential therapeutic strategy to improve intestinal barrier function and prevent the development of metabolic diseases.

## Introduction

Diet-related chronic diseases are the single largest cause of morbidity and mortality, afflicting>50% of the adult population. High fat high cholesterol containing Western diet-dependent changes in the genetic makeup of human gut microbiome are thought to underlie this epidemic of chronic diseases such as obesity, insulin resistance and atherosclerosis. Although diet-induced changes in gut bacteria *per se* are actively researched [1], a role for diet-induced changes of the gut itself is only beginning to be recognized. Gut microbe mediated changes in the metabolism of dietary components [2] and/or intestinal function is likely the underlying mechanism [3]. Association between circulating bacterial endotoxin lipopolysaccharide (LPS) and metabolic diseases has shifted the focus from actual bacterial infections in the etiology of these diseases to increased translocation of bacterial products (e.g., LPS) due to increase in intestinal permeability [4]. Functionally altered gut mucosa with increased permeability is seen in patients with chronic heart failure [5] and although the origin of chronic inflammation and increased levels of circulating cytokines in these patients is still unclear, role of small yet pathological amount of intestinally derived LPS in inducing systemic inflammation is increasingly being recognized [6]. Furthermore, uremia-induced disruption of colonic epithelial tight junction proteins and changes in intestinal permeability results in sustained systemic inflammation associated with chronic kidney disease [7] underscoring the importance of targeted improvement in intestinal barrier function as a potential therapeutic strategy. While synbiotic therapy has been shown to decrease bacterial translocation from the gut in a pilot study [8], attempts to recondition the barrier function through the use of so called ‘probiotics’, normally applied to the gut, have lower success in man than animals and are often incompatible with other pharmacological treatments [9]. For example, non-steroidal Anti-inflammatory Drugs (NSAIDs) cause a marked reduction in Lactobacilli and Bifidobacteria, which act in the maintenance of luminal pH, mucosal permeability, enterocyte adhesion, mucus production and immune system modulation, and are often used in probiotic therapy [10]. Furthermore, probiotic treatment also affects the metabolism of xenobiotics [11]. Plant-derived substances, or phytochemicals, e.g. curcumin can potentially offer similar effects as use of probiotics, although milder and yet free from these incompatible effects. The present study was undertaken to evaluate this strategy.

We examined whether “selective decontamination” of the gut by the use of non-absorbable antibiotics that are active in the intestinal lumen could attenuate the total release of bacterial LPS into circulation and thereby reduce chronic inflammation linked diseases namely Western-diet induced atherosclerosis and glucose intolerance. The combination of antibiotics used for standard selective decontamination (neomycin and polymyxin B) eliminates aerobic Gram-negative bacteria from the mucosal surfaces of the digestive tract, while the majority of the anaerobic flora persists and support colonization resistance [12]. These antibiotics are not absorbed and do not yield therapeutic serum concentrations in contrast to antibiotics which induce therapeutic serum concentrations (e.g., ciprofloxacin and cotrimoxazole) but are ineffective for “selective decontamination” of the gut [12]. It is hypothesized that by reducing the total amount of LPS produced within the gut, selective decontamination will attenuate Western diet-induced increase in LPS and downstream effect on intestinal barrier function. In addition we used a phytochemical Curcumin, the principal component of *Curcuma longa*, with demonstrated anti-inflammatory as well as anti-carcinogenic properties. Pharmacokinetics of curcumin and its poor systemic bioavailability suggest that it preferentially targets intestinal epithelial cells [13] and in limited studies has been shown to improve chemotherapy- [14] or oxidant-induced [15] intestinal barrier function.

Here we demonstrate that Western diet-induced increase in intestinal permeability leading to translocation of bacterial endotoxin LPS is reduced by selective decontamination of the gut via administration of non-absorbable antibiotics as well as by oral supplementation with Curcumin resulting in attenuation of inflammation-linked diseases namely atherosclerosis and glucose intolerance. This study establishes that targeted improvement of intestinal barrier function is a viable therapeutic strategy to attenuate Western diet-induced effects on intestinal mucosa, the resulting endotoxemia and development of associated diseases.

## Material and Methods

All chemicals were obtained from Sigma-Aldrich (St. Louis, MO) unless indicated otherwise.

### Animals, diets and study design

All animal procedures were approved by the Institutional Animal Care and Use Committees of Virginia Commonwealth University. LDL receptor knockout mice (LDLR−/−) originally obtained from Jackson Laboratory and maintained in the laboratory were used for all studies. Where indicated, at 10 weeks of age mice (littermates) were fed a high fat, high cholesterol Western type diet (WD, TD88137, Harlan Teklad) which contained 21% fat, 0.15% cholesterol, and 19.5% casein by weight with no sodium cholate for 16 weeks. In one set of studies, non-absorbable antibiotics (100 mg/L Neomycin and 10 mg/L Polymyxin B) were administered in drinking water; untreated or control mice received normal drinking water. In second set of studies, Curcumin (100 mg/kg in 0.5% carboxy methyl cellulose made fresh and administered within 15 minutes) was given as daily oral gavage; untreated or control mice were gavaged with vehicle alone. Administration of antibiotics or curcumin did not affect the food intake or WD-induced weight gain (Table 1).

**Table 1:** Body weights, plasma cholesterol and triglyceride levels.

Treatment	Body weight (g)	Total Cholesterol (mg/dl)	Total Triglyceride (mg/dl)
No Antibiotics	24.58±5.37	1570.08±531.81	267.50±35.32
With Antibiotics	28.68±6.20	1224.69±381.08	378.29±38.26
No Curcumin	26.25±1.86	1511.29±308.6	209.43±52.4
With Curcumin	23.72±4.04	1062.4±313.7	153.3±40.4

Prior to necropsy, mice were weighed and fasting plasma was collected. Total cholesterol and triglyceride levels were determined and shown as Mean ± SD, n = 6–10.

### Assessment of Intestinal barrier function

#### Plasma LPS

Mice were fasted overnight and at the time of necropsy, blood was collected by cardiac puncture and plasma was frozen at −80°C until analyzed. Plasma LPS levels were determined by Endpoint Chromogenic LAL (Lamilus Amebocyte Lysate) assay (Lonza, Walkersville, Maryland, USA) according to the manufacturer's instructions, with the following modifications: samples were diluted 5–10-fold to avoid interference with background color and preheated to 70°C for 10 minutes prior to analyses.

#### Intestinal alkaline phosphatase activity measurement

Intestinal alkaline phosphatase activity was measured as described [16]. Briefly, post-mitochondrial supernatant was assayed using p-nitrophenylphosphate as the substrate and appearance of p-nitrophenol monitored by measuring the absorbance at 405 nm. Specific enzyme activity was calculated as nmoles/h/mg protein used.

#### Translocation of FITC Dextran *in vivo*

Mice were orally gavaged with FITC-dextran 4 kDa (600 µg/kg body weight) dissolved in deionized water and plasma was collected after 4 h. Using three aliquots of plasma in duplicate, FITC fluorescence (excitation 488 nm and emission 518 nm) was measured using Perkin Elmer Victor 3 plate reader and concentration of FITC in plasma determined using a standard curve and expressed as ng/µl of plasma.

### Intraperitoneal glucose tolerance tests

10-week-old LDLR−/− mice were fed a Western diet for 16 weeks. After an overnight fast, a single bolus of glucose (2 mg/g body weight) was given intraperitoneally. Blood glucose levels were determined by commercially available glucometer using tail vein blood at 0, 15, 30, 60, 120 min.

### Assessment of atherosclerosis by *en face* analyses

The aorta was dissected from the heart to the iliac bifurcation, cleaned of any surrounding tissue, opened longitudinally, pinned on black wax and fixed for 24 h in 10% buffered formalin. The fixed aortas were imaged on a black background using a Canon Digital Camera fitted with a 60 mm, f/2.8 Macro Lens. Total area and the area occupied by the lesions in the aortic arch and total aorta was determined using AxioVision Image Analysis software as described earlier [17]. The person quantifying the area occupied by lesions was blinded to the identity of the images.

### Activation of macrophages

#### NF-κB driven luciferase expression

Thioglycollate elicited peritoneal macrophages were plated in 24-well plates. Medium was replaced after 4 h to remove non-adherent cells and cells were transduced with AdNF-κB-Luc (5 MOI) (Vector Labs, Burlingame, CA). After 24 h, cells were treated with 50 µM oleic acid [18] for 24 h followed by LPS (50 pg/ml) for 4 h. Luciferase activity was measured and normalized to cellular protein.

#### Secretion of cytokines and chemokines

Thioglycollate elicited peritoneal macrophages were plated in 24-well plates. Medium was replaced after 4 h to remove non-adherent cells and cells were treated with LPS (50 pg/ml) ±50 µM oleic acid for 24 h. Conditioned medium was used to determine the levels of secreted IL-6 and MCP-1 levels by ELISA (eBioscience, San Diego, CA).

### Assessment of permeability of intestinal epithelial cell monolayer

Caco-2 cells, obtained from the American Type Culture Collection (ATCC, Rockville, MD, USA), were plated on polycarbonate Transwells at 1×10^5^ cells/cm^2^ and grown in DMEM (Life Technologies, Carlsbad, CA) containing fetal calf serum (10% v/v), penicillin (100 U/ml) and streptomycin (100 mg/ml) for 21 days at 37°C in 5% CO_2_ in air. Prior to the start of the experiment the trans-epithelial electrical resistance (TEER) of the Caco-2 cultures was measured with an EVOM (World Precision Instruments, USA) to confirm their fully differentiated state. TEER is a measure of tight junction integrity and indicator of differentiation. Cultures displaying a TEER greater than 700 Ω cm^2^ were considered suitable for the study. Cells were pre-treated with 5 µM curcumin (in DMSO) for 48 h before stimulating with LPS (1 µg/ml) in serum free medium for 24 h.

#### Translocation of mannitol

After 24 hours, 1 ml [^14^C] Mannitol (0.55×10^5^ dpm; 0.49 µmole) (Perkin Elmer, Akron, OH) in HBSS was added to the apical side. Medium (50 µl, in duplicate) was collected from the basolateral side at 0.5, 1, 2 and 4 hours to measure paracellular permeability.

#### Translocation of FITC Dextran

After 24 h, 1mg/ml solution of FITC-Dextran in HBBS was added to the apical chamber and medium (50 µl, in duplicate) was collected from the basolateral side at 0.5, 1, 2 and 4 hours.

### Expression of tight junction proteins

The detergent insoluble fraction corresponding to the actin cytoskeleton-associated proteins was prepared as follows: Following indicated treatments, Caco-2 cell monolayers were washed with ice-cold HBSS and exposed for 10 minutes on ice to 500 µL of lysis buffer (1%Triton X-100, 5 mM EGTA in 50 mM Tris-HCl buffer pH 7.4 containing protease and phosphatase inhibitor cocktail). Cell lysates were centrifuged at 14,000 g for 5 min at 4°C to sediment the high-density actin-rich fraction. Proteins were separated on 4–20% gradient SDS-PAGE (BioRad, Hercules, CA) transferred to PVDF membrane (Millipore, Billereca MA) and used to determine the protein levels of ZO-1 and Claudin-1 using specific antibodies (Santa Cruz, Dallas TX) and normalized to β-actin.

### Statistical analyses

Comparison between untreated and treated groups was performed using Student's t-test and P<0.05 was considered significant. One way ANOVA with Bonferroni post-hoc correction was used for multiple comparisons and details are included in Figure legends.

## Results

### Western diet-induced release of endotoxin (LPS) due to enhanced intestinal permeability was attenuated by oral non-absorbable antibiotics or curcumin supplementation

**Figure 1:**
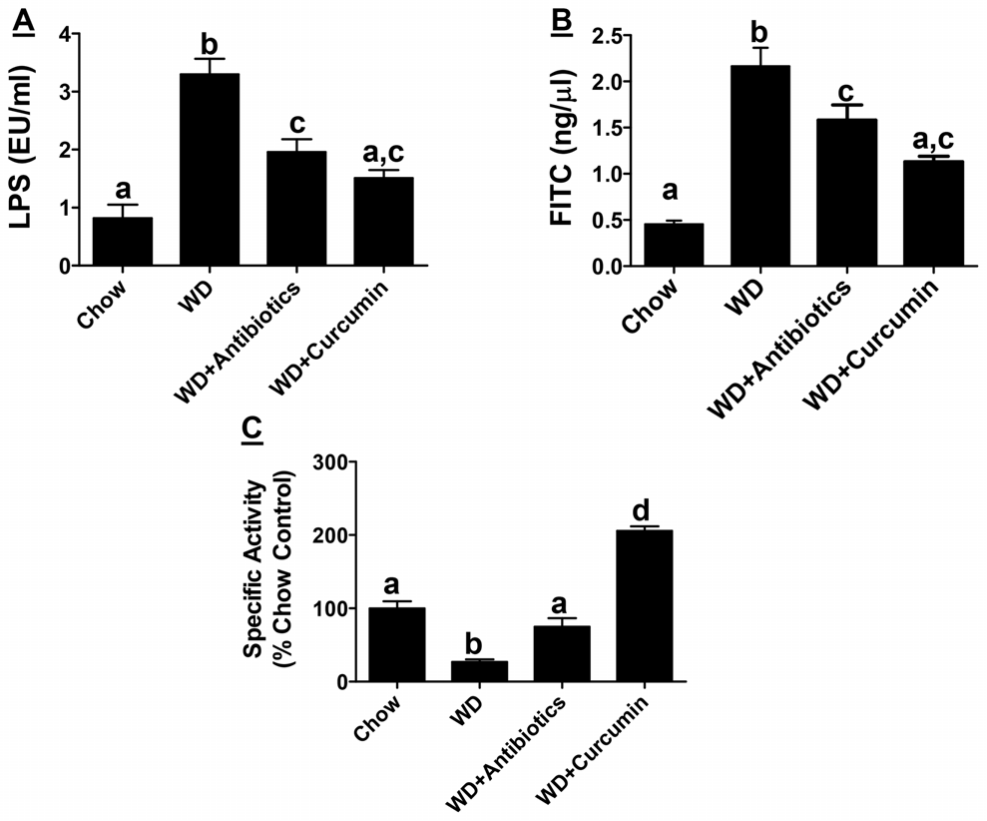
Supplementation with non-absorbable antibiotics or curcumin improves Western diet induced intestinal barrier dysfunction. Ten week old LDLR−/− mice were fed ad libitum High Fat High Cholesterol containing Western type diet (TD 88137, WD) for 16 weeks. Experimental groups either received non-absorbable antibiotics (100 mg/L Neomycin and 10 mg/L Polymyxin B) in drinking water or were gavaged daily with Curcumin (100 mg/kg in 0.5% carboxymethylcellulose). Panel A: Fasting plasma was collected at the time of necropsy and circulating LPS levels (EU/ml) were determined. Data are presented as Mean ± SEM, n = 6–9 per group. Panel B: Overnight fasted mice were gavaged with FITC-dextran 4 kDa and plasma samples were collected after 4 h. Appearance of FITC-dextran in plasma was monitored and the data (Mean±SEM, n = 6–9, per group) are presented as FITC concentration (ng/µl). Panel C: Intestinal Alkaline Phosphatase activity was determined and Specific activity (nmoles PNP released/h/mg protein) expressed as % chow-fed controls is shown (Mean ± SEM, n = 6–9 per group). P<0.0001 by One way ANOVA with Bonferroni post-hoc correction; Dissimilar letters above the bars indicate significant differences between groups (P<0.05).

A significant increase in plasma LPS levels (EU/ml) was induced by Western diet (0.82±0.77 vs 3.32±1.79) and this increase was attenuated in experimental groups receiving either non-absorbable antibiotics (1.90±0.91) or curcumin (1.51±0.39) (Figure 1A). LPS is continuously generated in the lumen of the intestine by the gut bacteria and an intact intestinal barrier prevents the release of even trace yet pathological amounts of LPS into the circulation. Translocation of FITC-dextran 4kDa to plasma compartment was monitored as an additional parameter to directly assess improvement in intestinal barrier function by antibiotics or curcumin. The appearance of orally administered FITC dextran (ng FITC/µl plasma) was significantly reduced by supplementation with non-absorbable antibiotics (2.17±0.53 vs 1.53±0.49) or curcumin (2.11±0.32 vs 1.13±0.15) further establishing an improvement in intestinal barrier function by these two interventions (Figure 1B). In addition to intestinal barrier function which restricts the release of LPS that is continuously produced in the intestinal lumen, intestinal alkaline phosphatase (IAP) associated with the brush border detoxifies LPS by dephosphorylation of the Lipid A moiety (the primary source of the endotoxic effects) and is considered as an important gut mucosal defense factor. Increased translocation of bacteria/bacterial products is observed in IAP−/− mice along with increased obesity [19] emphasizing the role of IAP not only in maintaining the intestinal barrier function but also in metabolic diseases. Western diet feeding significantly reduced IAP activity (∼↓75%) and this decrease was attenuated by supplementation with antibiotics (∼↓25%) (Figure 1C). Notably, curcumin supplementation significantly increased IAP activity compared to chow diet fed control mice (∼2 fold increase). These data suggest that oral supplementation with antibiotics or curcumin significantly improve intestinal barrier function and attenuate the release of LPS into the circulation.

### Low dose LPS activates NF-κB and increases secretion of pro-inflammatory cytokines/chemokines from macrophages

**Figure 2:**
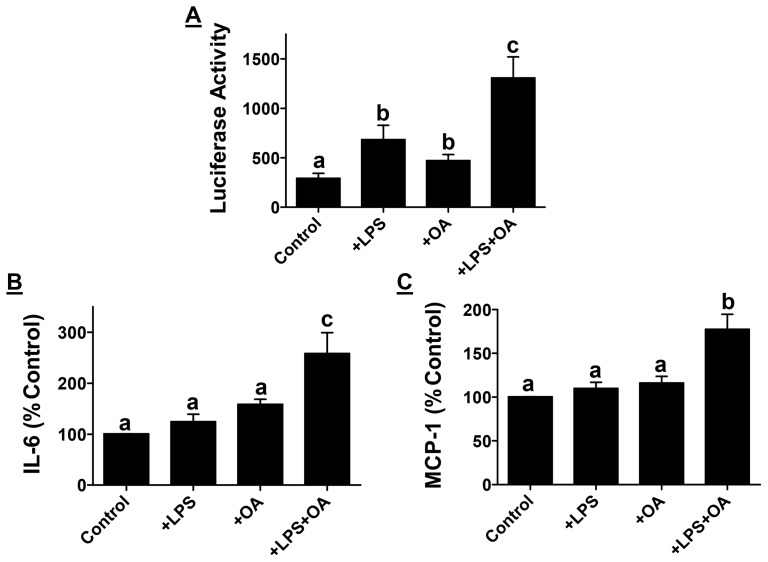
Increased macrophage activation by low dose LPS. Panel A: Mouse peritoneal macrophages were transduced with AdNF-κB-Luc and treated with LPS (50 pg/ml) or Oleic acid (50 µM) as indicated. Luciferase activity was normalized to cellular protein and data are presented as percent untreated Controls (Mean ± SEM, n = 6). Panel B and C: Conditioned medium was collected after incubation of mouse peritoneal macrophages with LPS with or without oleic acid as indicated and IL-6 and MCP-1 levels were determined by ELISA. Data are expressed as percent of no addition control (Mean ± SEM, n = 6). P<0.0001 by One way ANOVA with Bonferroni post-hoc correction; Dissimilar letters above the bars indicate significant differences between groups (P<0.05).

While effects of high doses of LPS, as seen in sepsis, on macrophage activation and subsequent production of pro-inflammatory cytokines are well established, consequences of low doses of LPS on macrophage activation and subsequent signaling is only beginning to be evaluated. Under hyperlipidemic/hypercholesterolemic conditions that prevail following consumption of Western diets, increased plasma lipids, especially free fatty acids, can further contribute to macrophage activation in cooperation with circulating LPS. To determine the contribution of such a mechanism in macrophage activation, we examined activation of pro-inflammatory transcription factor NF-κB in mouse peritoneal macrophages. Low dose LPS (50 pg/ml) significantly increased NF-κB-driven luciferase activity. Although exposure to oleic acid (major fatty acid in Western diet) also significantly increased NF-κB activation, the increase in luciferase expression in the presence of LPS along with oleic acid was significantly higher than that seen with LPS or oleic acid alone suggestive of synergism (Figure 2A). These data suggest that in the presence of Western diet derived oleic acid, trace amounts of intestinally derived LPS can significantly activate pro-inflammatory transcription factor NF-κB. Consequently, there was also a significant increase in the secretion of pro-inflammatory cytokine IL-6 (Figure 2B) and chemokine MCP-1 (Figure 2C) from macrophages exposed to LPS in the presence of oleic acid. Thus, Western diet feeding not only disrupts the intestinal barrier function releasing gut bacteria derived LPS into circulation that activates macrophages, but increased levels of circulating lipids under these conditions further exacerbate macrophage activation releasing pro-inflammatory chemokine/cytokines that can contribute to macrophage infiltration into tissues resulting in chronic inflammation and associated diseases.

### Oral supplementation with non-absorbable antibiotics or curcumin attenuates Western diet induced glucose intolerance and atherosclerosis

**Figure 3:**
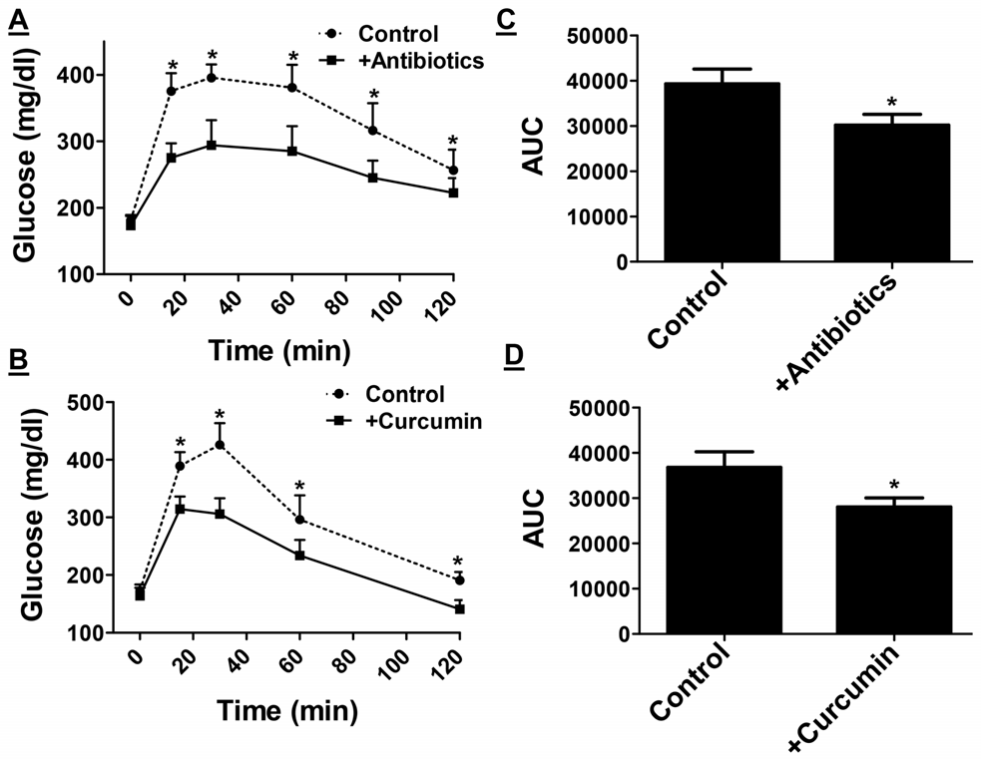
Western diet-induced Glucose intolerance is attenuated in mice receiving non-absorbable antibiotics or curcumin. Ten week old LDLR−/− mice were fed ad libitum High Fat High Cholesterol containing Western type diet (TD 88137) for 16 weeks with or without supplementation with non-absorbable antibiotics (Panels A and C) or curcumin (Panels B and D). Panels A and B: Intraperitoneal glucose tolerance tests were performed as described under Methods. Blood glucose levels at indicated times are shown (Mean ± SEM, n = 6). Panels C and D: Area under the curve was calculated using GraphPad Prism and data are presented as Mean ± SEM, n = 6. *P<0.05. One way ANOVA with Bonferroni post-hoc correction was also performed using Western diet fed mice as controls (P<0.018) but no significant difference (P>0.05) was observed between groups supplemented with either antibiotics or curcumin indicative of similar effects of these two treatments.

Consumption of Western type diets is associated with the development of glucose intolerance and/or heart disease. In order to examine the potential effects of antibiotics or curcumin supplementation on the development of these Western diet-induced metabolic diseases, intra-peritoneal glucose tolerance tests were performed after 16 weeks of feeding. Fasting glucose levels were not significantly different between control and treated mice. However, significant improvement in glucose tolerance was observed by antibiotic (Figure 3A) or curcumin supplementation (Figure 3B); the initial rise in blood glucose and levels at all-time points were significantly lower in mice given antibiotics or curcumin. Area under the curve, AUC, was calculated and a significant decrease was observed in mice treated with antibiotics (Figure 3C, 39400±8000 vs 30200±5800) or curcumin (Figure 3D, 36800±12000 vs 28000±7000).

**Figure 4:**
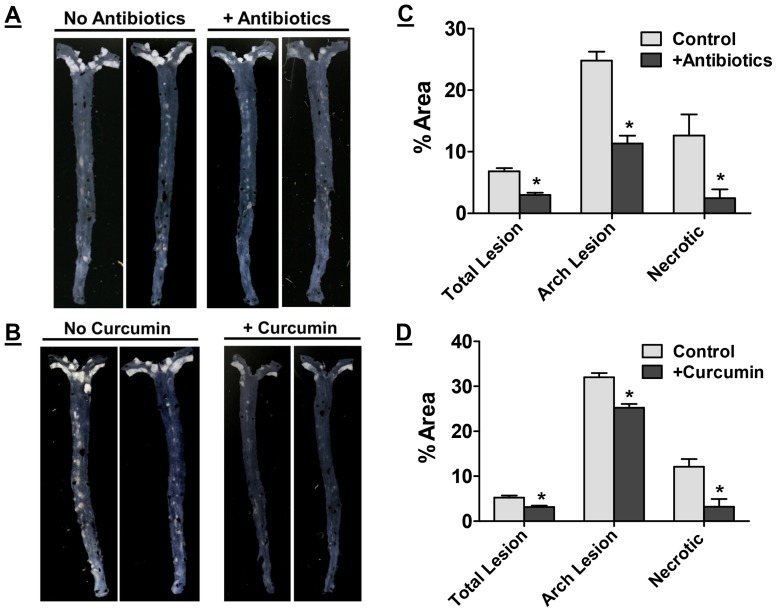
Supplementation with non-absorbable antibiotics or curcumin attenuates western diet induced atherosclerosis. Ten week old LDLR−/− mice were fed ad libitum High Fat High Cholesterol containing Western type diet (TD 88137) for 16 weeks with or without supplementation with non-absorbable antibiotics (Panels A and C) or curcumin (Panels B and D). Panels A and B: Aortas were isolated at the time of necropsy, cleaned of any adventitious tissue and imaged on a black background for en face analyses. Representative images are shown. Panel C and D: Area occupied by atherosclerotic lesions in the total aorta or aortic arch and necrotic area in aortic root lesions was quantified and shown as % Area (Mean ± SEM, n = 9–11 per group). *P<0.05. One way ANOVA with Bonferroni post-hoc correction was also performed using Western diet fed mice as controls (P<0.0001 for total and arch lesion area and P = 0.0013 for necrotic area) but no significant difference (P>0.05) was observed between groups supplemented with either antibiotics or curcumin indicative of similar effects of these two treatments.

Development of atherosclerosis was assessed by *en face* analyses and representative images are shown in Figure 4A and 4B. A dramatic reduction in the area occupied by atherosclerotic plaques was observed in mice receiving non-absorbable antibiotics or curcumin. Area occupied by the lesions was quantified and shown as percent of the total area. Supplementation with antibiotics (Figure 4C) or curcumin (Figure 4D) significantly reduced the area occupied by the lesions in the aortic arch (24.82±1.52 vs 11.34±1.26 and 32.02±0.94 vs 25.22±0.86, respectively) as well as entire aorta (6.82±0.5 vs 3.0±0.3 and 5.20±0.5 vs 3.14±0.3, respectively). It is noteworthy that supplementation with antibiotics or curcumin did not have any significant effect on plasma cholesterol or triglyceride levels (Table 1) consistent with the reported improvement in atherosclerosis with MyD88 and TLR4 deficiency in ApoE−/− background without significant changes in plasma lipids [20].

H&E stained aortic root sections were used to determine the necrotic area within each plaque. Increased macrophage inflammation is associated with necrosis of lesion associated macrophages. As shown in Figures 4C and 4D, a significant decrease in necrotic area was noted in antibiotic or curcumin treated mice indicating reduced macrophage necrosis.

### Improvement in intestinal barrier function is the underlying mechanism of the observed effects of non-absorbable antibiotics and curcumin

**Figure 5:**
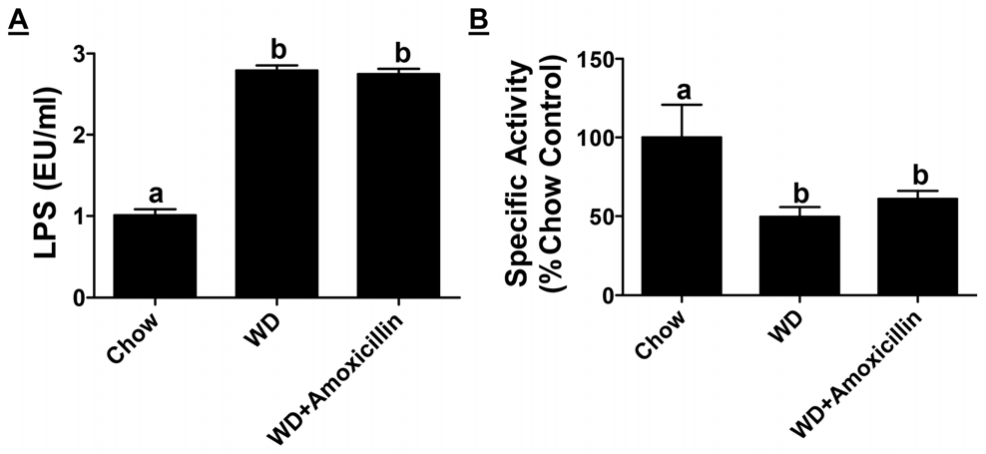
Effect of absorbable antibiotic Amoxicillin on Western diet induced changes in intestinal barrier function. Ten week old LDLR−/− mice were fed ad libitum High Fat High Cholesterol containing Western type diet (TD 88137) for 16 weeks with or without supplementation with amoxicillin in drinking water. Panel A: At the time of necropsy, fasting plasma was collected and LPS levels were determined. Data are presented as Mean ± SD, n = 6. Panel B: Intestinal Alkaline Phosphatase activity was determined and Specific activity (nmoles PNP released/h/mg protein) expressed as % chow-fed controls is shown (Mean ± SEM, n = 6).

**Figure 6:**
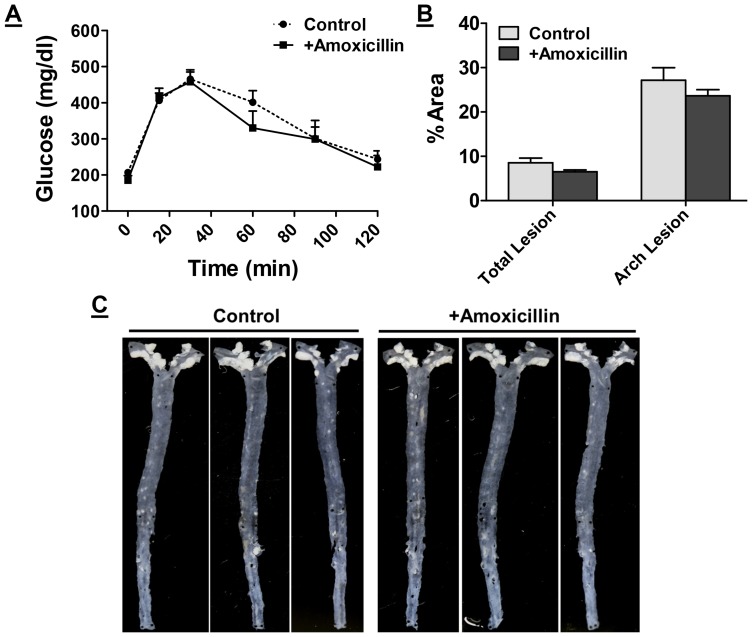
Effect of absorbable antibiotic Amoxicillin on Western diet induced development of glucose intolerance and atherosclerosis. Ten week old LDLR−/− mice were fed ad libitum High Fat High Cholesterol containing Western type diet (TD 88137) for 16 weeks with or without supplementation with amoxicillin in drinking water. Panel A: Intraperitoneal glucose tolerance tests were performed as described under Methods. Blood glucose levels at indicated times are shown (Mean ± SEM, n = 6). Panel B: Aortas were isolated at the time of necropsy, cleaned of any adventitious tissue and imaged on a black background for en face analyses. Representative images are shown. Panel C: Area occupied by atherosclerotic lesions in the total aorta or aortic arch was quantified and shown as % Area (Mean ± SEM, n = 6).

To further establish that intestine is the target organ for the beneficial effects of non-absorbable antibiotics as well as curcumin, LDLR−/− mice on Western diet were given absorbable antibiotics (Amoxicillin 100 mg/L) in drinking water. Amoxicillin is rapidly absorbed (>1 h transit time in the intestine) and is likely to have limited intestinal effects. Supplementation with amoxicillin did not significantly affect Western-diet induced increase in plasma LPS levels (Figure 5A) demonstrating a negligible effect on intestinal barrier function. Furthermore, no change in IAP activity was noted with amoxicillin supplementation (Figure 5B) confirming the intestine-localized effect of selective decontamination. Consistently, supplementation with amoxicillin neither improved Western diet-induced glucose intolerance (Figure 6A) nor had any significant effect on atherosclerotic lesion development (Figure 6BD and 6C). These data are consistent with limited effects observed in clinical trials evaluating the use of antibiotics (e.g., Azithrimycin) in reducing the risk for heart disease [21] and also provide additional support for the role of intestinal barrier function in the development of chronic inflammatory diseases.

### Curcumin improves intestinal barrier function

**Figure 7:**
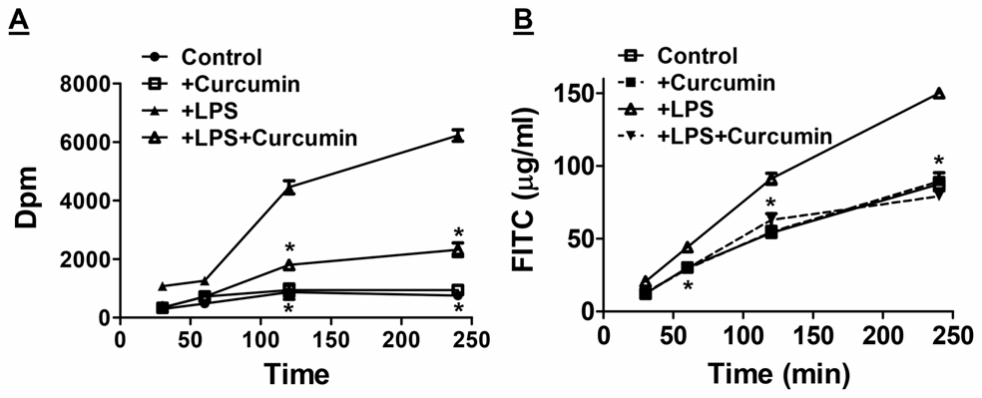
Curcumin attenuates LPS-induced increase in intestinal epithelia cell permeability. Panel A: Fully differentiated Caco-2 cell monolayers in Transwell inserts were treated with LPS or curcumin and transport of [^3^H]-mannitol (expressed as dpm, Mean ± SEM, n = 6) to the bottom chamber was monitored at indicated time points. Panel B: Improvement of LPS induced intestinal epithelial cell permeability by curcumin was also assessed by monitoring the transport of FITC dextran 4 kDa across Caco-2 cell monolayer. Appearance of FITC dextran in the bottom chamber at indicted time points was determined and expressed as µg/ml. *P<0.05 compared to +LPS.

Currently no specific therapeutic strategies are available to directly restore intestinal barrier function. Use of probiotics is proposed, however, limited ability to be administered along with other pharmaceuticals has been the major hurdle. Data presented above identifies curcumin as an eco-biological phytochemical that significantly restores Western diet-induced dysfunction of intestinal barrier function. To further examine the direct effects of curcumin on intestinal epithelial cells, Transwell-grown monolayers of intestinal epithelial Caco-2 cells were exposed to LPS (to disrupt barrier function) with or without pre-treatment with curcumin and permeability of the monolayers determined by monitoring the translocation of [^3^H]-mannitol or FITC dextran 4 KDa from the apical to the basal side. Time dependent increase in the translocation of [^3^H]-mannitol (Figure 7A) and FITC dextran (Figure 7B) was induced following exposure to LPS. Although curcumin did not have any effect on the basal or unstimulated translocation, it significantly reduced LPS-induced translocation of [^3^H]-mannitol as well as FITC dextran. These data establish the direct effects of curcumin in preventing LPS-induced disruption of intestinal epithelial cell barrier permeability.

**Figure 8:**
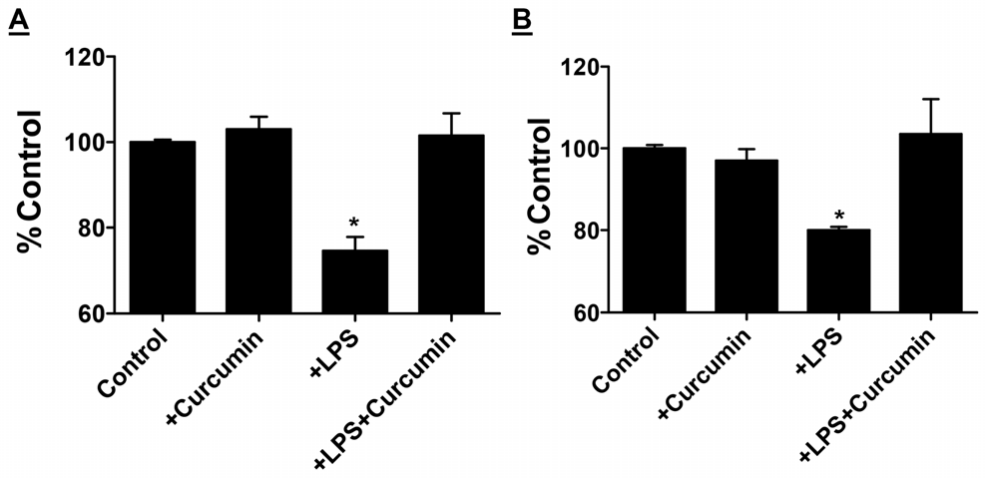
Protection from LPS induced intestinal epithelia cell dysfunction by Curcumin. Caco-2 cell monolayers were treated with LPS or curcumin as indicated and detergent insoluble fractions were analyzed by Western Blot analyses. Expression levels of ZO-1 (Panel A) or Claudin-1 (Panel B) were normalized to β-actin and presented as percent of untreated controls. *P<0.05 compared to untreated controls.

Intestinal epithelium maintains its selective barrier function through the formation of network of tight junction proteins such as claudins and ZO-1. These proteins interact intracellularly with adaptor proteins that link to the actin cytoskeleton [22] and maintenance of intestinal barrier function is dependent on the expression of tight junction protein associated with cytoskeletal protein fraction or the detergent insoluble protein fraction [23]. When Caco-2 cells were exposed to LPS, there was 26% and 17% decrease in detergent insoluble actin-associated ZO-1 (P = 0.01) and claudin 1 expression (P = 0.05) respectively. This LPS-induced reduction in expression was prevented in the presence of curcumin (Figure 8A and 8B); curcumin alone in the absence of LPS had no effect on ZO-1 or claudin 1 expression. Taken together with effects of curcumin in reducing the translocation of [^3^H]-mannitol and FITC in LPS-treated Caco-2 cells as well as attenuation of LPS release in vivo, these data provide strong evidence that curcumin can beneficially affect intestinal barrier function.

## Discussion

**Figure 9:**
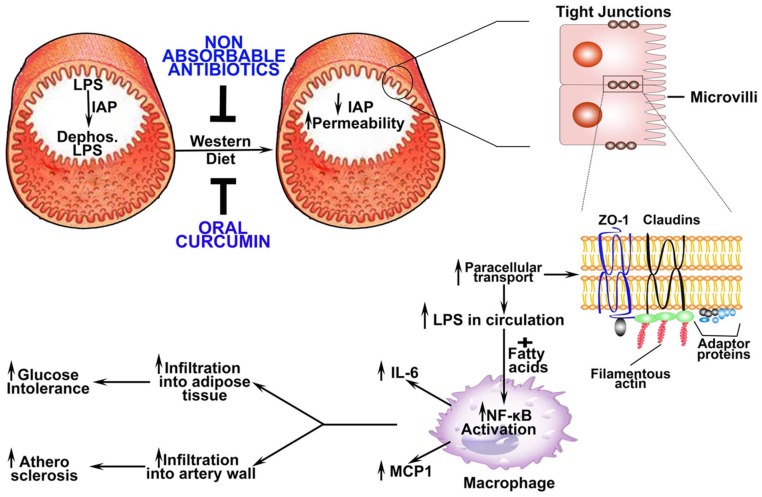
Proposed model for attenuation of Western diet induced changes in intestinal barrier dysfunction and subsequent development of glucose intolerance and atherosclerosis by non-absorbable antibiotics or curcumin. Under normal physiological conditions, intestinal barrier restricts the release of luminal LPS from gut bacteria into circulation. Intestinal alkaline phosphatase (IAP) is one of the major components of this “barrier” and it detoxifies LPS by dephosphorylation of the Lipid A moiety. Consumption of Western diet decreases IAP (Figure 1B) thereby increasing the effects of luminal LPS on intestinal epithelial cells and increasing intestinal permeability (Figure 1C). Luminal LPS-induced decrease in the expression of tight junction proteins ZO-1 and Claudin -1 (Figure 3G) increases the paracellular transport of LPS into circulation (Figure 1A). Trace yet pathological doses of LPS along with Western diet-induced increase in circulating lipids (e.g., fatty acids) enhance macrophage activation and induce NF-κB-driven gene expression (Figure 3A) resulting in increased secretion of pro-inflammatory cytokines and chemokines (Figure 3C and D). Increased infiltration of activated macrophages into adipose tissue or artery wall leads to the development of glucose intolerance and atherosclerosis. Administration of non-absorbable antibiotics or curcumin prevents Western diet-induced disruption of intestinal barrier function and thereby attenuates these sequential events resulting in attenuation of Western diet-induced glucose intolerance and atherosclerosis (Figure 2).

The findings reported in this study not only demonstrate Western diet-induced changes in intestinal barrier function (reduction in IAP and tight junction proteins resulting in increased permeability) leading to increased endotoxemia, macrophage activation and subsequent development of glucose intolerance and atherosclerosis but also establish that improvement of this barrier function can significantly attenuate this pathological sequelae (Figure 9). While selective gut decontamination by the use of non-absorbable antibiotics provides the “proof of concept” that reduction in total intestinal bacterial content could be beneficial in preventing Western diet-induced metabolic diseases, the significant reduction in glucose intolerance as well as atherosclerosis by oral curcumin demonstrates the importance of targeted improvement in intestinal barrier function as a potential therapeutic strategy. This represents a change in the existing paradigm and places the focus on improving intestinal barrier function rather than direct modulation of gut bacteria itself.

Intestinal barrier is a multi-layer defense system [24] consisting of lumen as the first line of defense where enzymatic degradation of bacteria and antigens occurs and the commensal bacteria prevent colonization of pathogens. The mucus layer prevents the adherence of bacteria to the epithelial cells and reduces direct bacterial-epithelial interactions. Tight junctions between the epithelial cells restrict para-cellular transport of luminal contents. Intestinal Alkaline Phosphatase (IAP) is part of the luminal first line of defense and catalyzes the removal of one of the two phosphate groups from the toxic lipid A moiety of LPS producing monophosphoryl-LPS that still binds to TLR4 but predominantly acts as an TLR4 antagonist [25]. We observed attenuation of western-diet induced decrease in IAP activity by non-absorbable antibiotics and an increase by curcumin. Tuin et al have reported a decrease in IAP in patients with inflammatory bowel disease [26] and heat stable, chimeric human alkaline phosphatase is currently being evaluated as a protein therapeutic for gut dysbioses, inflammatory bowel disease and acute kidney injury [27]. Restoration of Western diet-induced decrease in IAP activity by selective gut decontamination as well as with curcumin supplementation shown here strongly suggests a potential therapeutic use of this strategy for multiple diseases. In this regard, it is noteworthy that we have earlier demonstrated significant improvement in chronic kidney disease by curcumin supplementation [28], [29]. The two interventions examined in this study also decreased para-cellular transport and prevented western diet-induced reduction in the expression of tight junction proteins (ZO-1 and Claudin-1). Taken together, these data provide direct evidence for improvement in the intestinal barrier function by these two interventions at multiple levels namely luminal detoxification of LPS by IAP and paracellular transport of LPS by modulating expression of tight junction proteins.

Low grade chronic inflammation is central to the development of obesity and Western diet-induced metabolic diseases and trace, yet pathological, amounts of intestine-derived LPS released into the circulation accompanies many of these conditions [30]. Molecular mechanisms underlying the effects of such low grade endotoxemia are not completely understood. Maitra et al have recently described alternate signaling and activation of macrophages in vitro by exposure to low doses of LPS [31]. While exposure to high doses of LPS prevents response to later stimulation due to LPS-induced internalization of surface TLR4 (endotoxin tolerance), exposure to low doses of LPS exaggerates the response to subsequent exposure to TLR4 ligands (priming) [32], underscoring the importance to low grade endotoxemia in exacerbation of macrophage inflammatory responses. Circulating free fatty acids represent additional TLR4 ligands during dyslipidemia caused by the consumption of Western type diet and the data presented here demonstrates more than additive effect of LPS and oleic acid on macrophage activation suggesting that this diet contributes to low grade chronic inflammation by first increasing the release of gut-derived LPS into circulation that can prime the macrophages and secondly by increasing the circulating free fatty acids that can further exacerbate this inflammatory response. While infiltration of activated macrophages into adipose tissue and resulting adipose tissue inflammation underlie the development of insulin resistance and diabetes, macrophage infiltration into artery wall and conversion to lipid laden foam cells leads to the development of atherosclerosis (Figure 9).

Curcumin, a polyphenolic natural compound and a principal ingredient of Asian spice Turmeric is reported to possess anti-inflammatory and anti-oxidant properties. However, the mechanisms underlying its observed effects have not been completely understood. Dietary curcumin is poorly absorbed and undergoes glucuronidation and excretion limiting its release into the circulation. Consequently, significant efforts are currently being directed to the development of novel methods for systemic or targeted delivery of curcumin [33]. The data presented here demonstrate for the first time that curcumin exerts potent effects in reducing metabolic diseases such as diabetes and atherosclerosis by modulating the intestinal barrier function, precluding the need for effective absorption and systemic bioavailability. Curcumin-mediated reduction in ox-LDL uptake by THP-1 cells in vitro and reduction in inflammatory cytokines in vivo were thought to be the mechanisms underlying the observed decrease in atherosclerosis [34]. Based on the observed reduction in monocyte adhesion to curcumin treated endothelial monolayers in vitro, Coban et al speculated that reduced macrophage infiltration is likely the underlying mechanism for the observed reduction in atherosclerosis by dietary curcumin [35]. In a recent Phase II trial to evaluate the efficacy of oral curcumin for pancreatic cancer, despite an oral dose of 8 g/day, negligible amounts of free curcumin was detected in the plasma and total curcumin levels (free + glucuronated/sulfated) were in the range of ng/ml, significantly lower than the dose used in most in vitro studies [36]. While low bioavailability may have thus far precluded the development of curcumin as a potent anti-inflammatory or anti-cancer agent, the current study provides a novel mechanism of action of curcumin. It is noteworthy that a recent pilot clinical study for cancer chemoprevention has demonstrated the safety of oral curcumin for long term use [37] paving the way for future studies to evaluate the efficacy of this phytochemical for attenuation of Western diet-induced metabolic diseases.

In conclusion, the data presented here provide direct evidence for the role of Western diet-induced disruption of intestinal barrier function in the development of metabolic diseases such as diabetes and atherosclerosis. Furthermore, these studies also identify curcumin as an agent effective in restoring the intestinal barrier function by modulating multiple components of this barrier including IAP and paracellular permeability. Future studies will examine the effects of curcumin on other components of the intestinal barrier to further advance our current understanding and to develop curcumin as a dietary supplement to attenuate multiple inflammation-linked diseases.
